# Obstetrical Superstitions

**Published:** 1907-06

**Authors:** 


### OBSTETRICAL SUPERSTITIONS.

Kissinger says that in all branches of medicine one meets with many curious superstitions. We, as a nation, are the least superstitious of any people, because we are the best educated people in the world. In obstetrics one meets with some curious ideas that have existed for centuries. The doctor knew of a case in which the mother and nurse positively refused to allow the doctor to weigh the baby because it was unlucky. The custom of burning the cloth used to dress the cord originated in a superstition. Experience has shown it to be of value, and science tells us why. It is considered unlucky to be born on Friday. The months and phases of the moon also influence the future of the baby. Galen said that "animals born when the moon is falciform are weak, feeble, and short lived, while those born when the moon is full are the reverse." On the other hand, it is an extreme good fortune to be the seventh daughter of a seventh son. To be born with the caul is also a rare good fortune. These membranes are dried and are supposed to possess a wonderful charm. The possessor is endowed with eloquence and is enabled to avoid many serious dangers. It is reported that these "cauls" may be bought in France for a reasonable price. A small drink of urine is advocated by some as an excellent aid to delivery. Holding a palm-leaf in her hand or placing a sardonyx between her breasts will do the same thing.—Pennsylvania Medical Journal.

